# Early veno-venous haemodiafiltration for sepsis-related multiple organ failure

**DOI:** 10.1186/cc3886

**Published:** 2005-11-09

**Authors:** Bernard Page, Antoine Vieillard-Baron, Karim Chergui, Olivier Peyrouset, Anne Rabiller, Alain Beauchet, Philippe Aegerter, François Jardin

**Affiliations:** 1 Medical Intensive Care Unit, University Hospital Ambroise Paré, Assistance Publique Hôpitaux de Paris, 9 avenue Charles de Gaulle, 92104 Boulogne, France; 2 Department of Biostatistics, University Hospital Ambroise Paré, Assistance Publique Hôpitaux de Paris, 9 avenue Charles de Gaulle, 92104 Boulogne, France

## Abstract

**Introduction:**

We conducted a prospective observational study from January 1995 to December 2004 to evaluate the impact on recovery of a major advance in renal replacement therapy, namely continuous veno-venous haemodiafiltration (CVVHDF), in patients with refractory septic shock.

**Method:**

CVVHDF was implemented after 6–12 hours of maximal haemodynamic support, and base excess monitoring was used to evaluate the improvement achieved. Of the 60 patients studied, 40 had improved metabolic acidosis after 12 hours of CVVHDF, with a progressive improvement in all failing organs; the final mortality rate in this subgroup was 30%. In contrast, metabolic acidosis did not improve in the remaining 20 patients after 12 hours of CVVHDF, and the mortality rate in this subgroup was 100%. The crude mortality rate for the whole group was 53%, which is significantly lower than the predicted mortality using Simplified Acute Physiology Score II (79%).

**Conclusion:**

Early CVVHDF may improve the prognosis of sepsis-related multiple organ failure. Failure to correct metabolic acidosis rapidly during the procedure was a strong predictor of mortality.

## Introduction

Septic shock is usually accompanied by acute renal injury, heralded by a drop in diuresis. However, when standard intermittent haemodialysis (IHD) is used to treat renal failure, the initiation of renal replacement therapy is often delayed by concerns about haemodynamic tolerance. With the availability of continuous veno-venous haemofiltration, a safe procedure in haemodynamically unstable patients [1], there is no reason to delay renal replacement therapy [2]. Moreover, haemofiltration has been reported to improve cardiopulmonary function in septic patients, even if they are not oliguric [3,4].

Based on these findings, in January 1995 we began to treat sepsis-related multiple organ failure with early continuous veno-venous haemofiltration combined with continuous veno-venous haemodiafiltration (CVVHDF). This strategy was our standard clinical practice for 10 years (from January 1995 to December 2004) and was accepted as a routine procedure by the Ethics Committee of the Société de Réanimation de Langue Française (Paris, France). Our clinical results are presented in this report, which focuses on the relation between rapid correction of metabolic acidosis with early renal replacement therapy and mortality.

## Materials and methods

### Patients

Between January 1995 and December 2004, all patients meeting at the same time criteria for sepsis, refractory circulatory failure, acute renal injury, and acute lung injury were included in the study, and data were prospectively collected for later analysis. Sepsis was defined as at least two of the following conditions occurring within the context of infection: temperature above 38°C or below 36°C, heart rate above 90 beats/minute, and white blood cell count above 12,000 or below 4,000 cells/mm^2 ^[5]. The causative bacterial agent was subsequently identified based on positive culture (blood or a sample from a localized site of infection) in 70% of cases. Refractory circulatory failure was defined as a persistent or growing metabolic acidosis despite adequate vasoactive support over an observation period of 6–12 hours, and was judged to be present if there was a base excess below -5 mmol/l at the end of this period. Acute renal injury was defined as a urinary output below 30 ml/hour during the period of observation [6]. Finally, acute lung injury was defined as an arterial oxygen tension/fractional inspired oxygen ratio below 300 mmHg and need for mechanical ventilation. However, patients meeting inclusion criteria but with a rapidly fatal underlying medical condition (McCabe score 2) were excluded.

### Haemodynamic monitoring and initial management of circulatory failure

Arterial pressure was monitored using an indwelling radial artery catheter, and central venous pressure was monitored using an internal jugular venous catheter. All patients had hypotension at admission (primary shock) or exhibited acute hypotension during their stay in the unit (secondary shock), defined as an arterial systolic pressure lower than 90 mmHg, as determined by invasive monitoring. This hypotension persisted despite aggressive fluid challenge and required continuous noradrenaline (norepinephrine) infusion. Fluid resuscitation was performed by administering 10–20 ml/kg plasma expanders (6% Hetastarch) over 30 minutes, followed by administration of enough crystalloids to achieve a central venous pressure of 12 mmHg or greater rapidly. Continuous infusion of noradrenaline was started at 0.1 μg/kg per minute and was progressively increased until a systolic radial pressure above 90 mmHg was achieved. Bedside echocardiography was used to measure cardiac index (using the Doppler technique) and left ventricular ejection fraction, as previously described [7]. Dobutamine was added at 5 μg/kg per minute when left ventricular ejection fraction was found to be lower than 40% on transthoracic or transoesophageal bedside echocardiography [7]. In cases in which circulatory improvement was judged to be insufficient with this combination (i.e. persistent and severe left ventricular systolic dysfunction by echocardiography), dobutamine was replaced by adrenaline (epinephrine) infusion at 0.5–2 μg/kg per minute [7].

### Additional therapies

All of the patients also required mechanical ventilation because of associated acute lung injury or acute respiratory distress syndrome, which was an inclusion criterion. Our strategy of low-stretch mechanical ventilation was reported previously [8]. In addition, 17 patients from the group received low-dose corticosteroids. This additional therapy was systematically used in our unit from January 2002, as has been recommended in the management of septic shock [9]. No patients received drotrecogin alfa (activated). Finally, all patients were given antibiotics in consultation with a microbiologist, according to culture findings.

### Severity indices

In all patients, a general severity index (the Simplified Acute Physiology Score [SAPS] II [10]) was calculated at admission. An organ dysfunction index (the Logistic Organ Dysfunction Score [11]) was calculated at admission (primary shock) or at the onset of circulatory failure (secondary shock). We also calculated the probability of hospital mortality (predicted mortality) using SAPS II and the 'standardized mortality ratio' by dividing the observed by the predicted hospital mortality. The severity of the patient's underlying medical condition was stratified using the McCabe score [12] as nonfatal (score 0) or ultimately fatal (score 1). As stated above, patients with a rapidly fatal underlying medical condition (McCabe score 2) were excluded. We also noted the presence or absence of a condition known to be associated with immunological incompetence.

### Veno-venous haemodiafiltration

CVVHDF was considered at the end of the observational period if a growing metabolic acidosis was observed, as defined above. Each CVVHDF session was performed using a Prisma pump (Hospal, Lyon, France). For vascular access, a double lumen catheter (Mahurkar, 11.5 Fr; Tyco Healthcare Group, Mansfield, MA, USA) was inserted percutaneously into either the right internal jugular vein or the femoral vein using the Seldinger technique. Blood flow was driven at 150 ml/minute through the polyacrylonitrile haemofilter (AN 69; Hospal). Ultrafiltration was maintained at 2,000 ml/hour. The ultrafiltrate was replaced by bicarbonate-buffered haemofiltration fluid (Hemosol B0; Hospal). Dialysate (bicarbonate 32 mEq/l; Hemosol B0; Hospal) flow rate was maintained at 1,000 ml/hour. The anticoagulant was intravenous heparin, with an initial bolus of 2,000–3,000 IU, followed by 300 IU/kg per day to maintain the patient's activated clotting time at 60–70 s.

Blood glucose checks were performed regularly during the procedure, and both hyperglycaemia (sometimes present in septic patients) and hypoglycaemia (related to the procedure) were immediately corrected.

Each session of CVVHDF lasted 3 days, with the same filter, and was followed by 2 or 3 days without CVVHDF. In five cases, however, early clotting after 12–24 hours required an immediate change in filter in order to follow the 3-day protocol. CVVHDF was then repeated for 3 days if necessary, until renal recovery. During the first session of CVVHDF a strictly neutral fluid balance was maintained, using the digital balance included in the Prisma pump. During the following sessions, a negative fluid balance was instituted if necessary.

### Statistical analysis

Statistical calculations were performed using the Statgraphics plus package (Manugistics, Rockville, MD, USA). Data are expressed as mean ± standard deviation or (in figures) as box and whisker plot analyses. Between group comparisons were performed using the χ^2 ^test with Yates' correction for categorical variables, and with the Mann–Whitney *U* test for continuous variables. A Wilcoxon signed rank test was used to compare paired variables. Cumulative survival curves were compared using the log rank test. Linear regression analysis was also performed when required.

## Results

**Figure 1:**
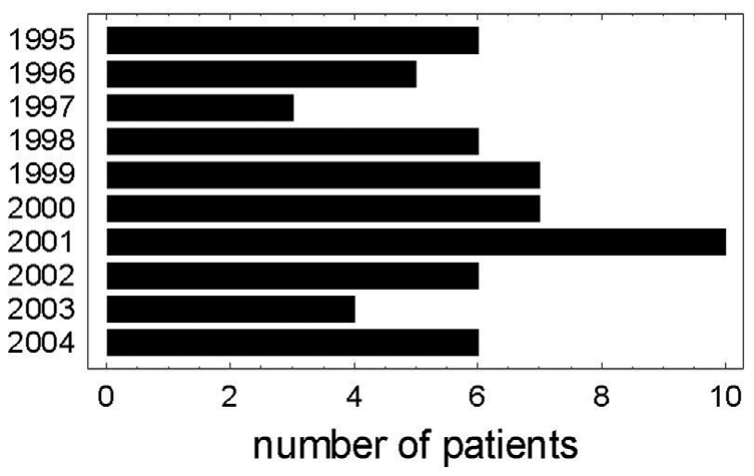
Number of patients included per year during the 10-year period of observation.

The study group included 38 men and 22 women, whose mean age was 57 ± 16 years. The average measured body weight was 72 ± 13 kg. Circulatory failure was present at admission in 44 patients (primary shock) or occurred after several days of hospitalization in our unit in 16 patients (secondary shock). The number of patients included per year during this 10-year period is presented in Figure 1. These patients were predominantly medical (45 medical patients versus 15 surgical patients). The causative bacterial agent was subsequently identified from a positive blood culture or from a localized site of infection in 42 cases; Gram-positive and Gram-negative agents were responsible in 22 and 20 patients, respectively. The average SAPS II score for the whole group was 67 ± 18. During the same period, 35 patients who met the inclusion criteria but with a rapidly fatal underlying disease (McCabe score 2) were excluded from the study.

**Figure 2:**
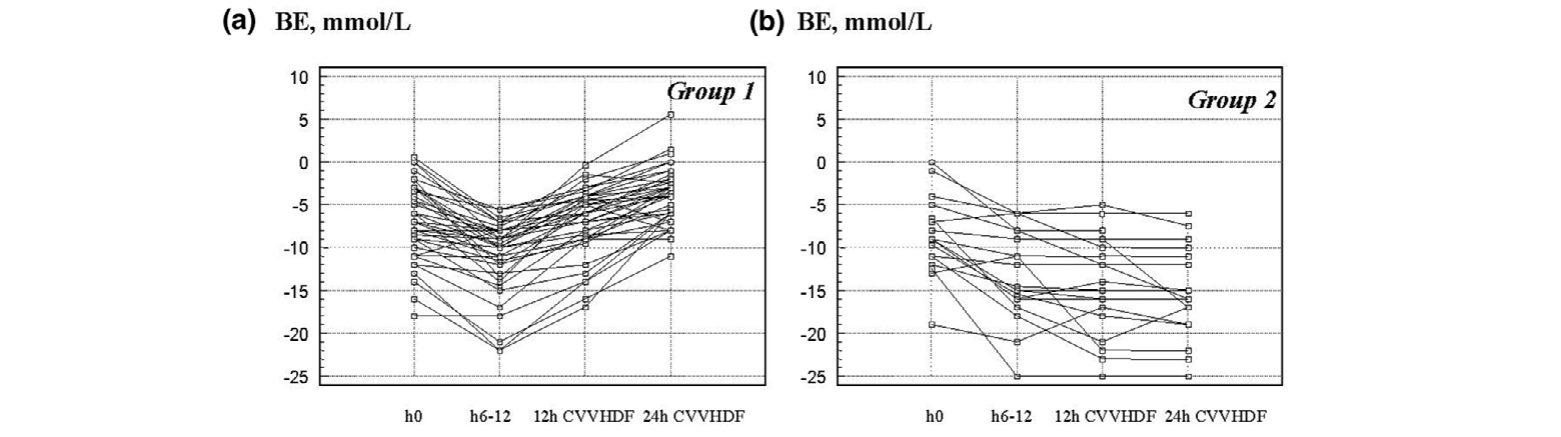
Individual changes in base excess. Shown are individual changes in base excess in **(a) **responders (group 1) and **(b) **nonresponders (group 2) over the 6- to 12-hour observation period (h 0 to h 6–12) and during the first 24 hours of CVVHDF. CVVHDF begins at h 6–12 on the x-axis; the patients have undergone 12 hours of CVVHDF at h 12 on the x-axis; finally, the patients have undergone 24 hours of CVVHDF at h 24 on the x-axis. CVVHDF, veno-venous haemodiafiltration.

**Figure 3:**
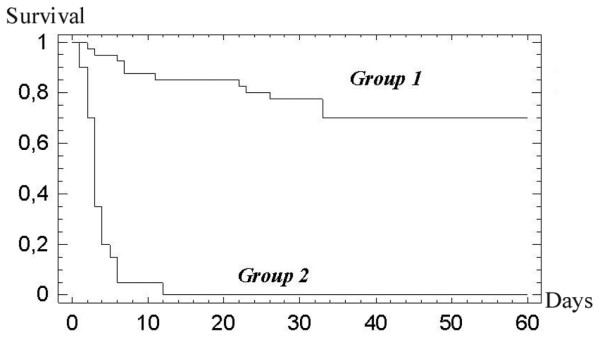
Cumulative survival. Shown are cumulative survival curves in group 1 (responder) and group 2 (nonresponder) patients, showing better outcome in group 1 (*P *< 0.0001, log rank test).

Individual changes in base excess during the observational period, defining refractory circulatory failure, are shown in Figure 2, and the trend in base deficit after 12 hours of CVVHDF was used to separate patients into two groups. In 40 responders (group 1), metabolic acidosis was reduced by the first 12 hours of CVVHVD. In 20 nonresponders (group 2), metabolic acidosis was unchanged or even deteriorated after 12 hours of CVVHDF (Figure 2). Table 1 summarizes the main physiological data for both groups. No between-group difference was observed apart from a significantly higher Logistic Organ Dysfunction Score in group 2. Mortality rate was 30% in group 1 and 100% in group 2 (*P *< 0.0000; Table 1 and Figure 3).

Twenty-eight patients recovered and 32 ultimately died, leading to a crude mortality rate of 53% for the whole group. This mortality rate was significantly lower than that predicted by SAPS II (79%; *P *= 0.01) leading to an standardized mortality ratio of 0.67.

### Metabolic acidosis

As stated above, metabolic acidosis was judged to be present if there was a base excess below -5 mmol/l at the end of the period of observation. All patients studied exhibited an increased blood lactate level at the end of the period of observation (5 ± 3 mmol/l, range 1.5–16.4 mmol/l). Individual blood lactate values were inversely and significantly correlated with individual base excess values (r = -0.61; *P *< 0.001). Plasma anion gap was calculated as follows: (sodium + potassium) – (chloride + bicarbonate). Our laboratory's normal value for anion gap is 16 mmol/l, and all patients studied except for one exhibited a widened anion gap (25 ± 6 mmol/l, range 11.6–43.8 mmol/l). Individual values for anion gap were inversely and significantly correlated with individual values for base excess (r = -0.62; *P *< 0.001). Plasma phosphate was also significantly increased (2.1 ± 0.9 mmol/l, range 0.6–4.4 mmol/l), and we also found a significant inverse correlation between individual values of plasma phosphate and base excess (r = -0.48, *P *= 0.002).

Average plasma electrolyte concentrations for both groups measured before CVVHDF implementation are presented and compared in Table 2.

### Refractory circulatory failure

**Figure 4:**
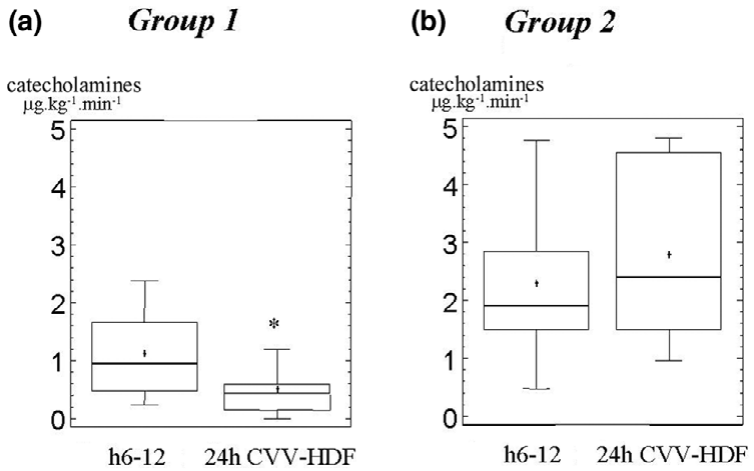
Changes in the amounts of catecholamines required. Shown are box and whisker plot analyses (median = horizontal line inside the box; mean = point inside the box) of changes in the amount of catecholamines required at onset of CVVHDF (h 6–12 on the x-axis) and after 24 hours of the procedure (h24 on the x-axis) in **(a) **group 1 (responders) and **(b) **group 2 (nonresponders). A significant reduction in need for catecholamines was observed in group 1 during CVVHDF (*P < 0.001). CVVHDF, veno-venous haemodiafiltration.

During the period of observation, haemodynamic support involved noradrenaline infusion alone in 28 patients, noradrenaline combined with dobutamine in 11 patients, and adrenaline in 21 patients. Average dosages of major catecholamines (for example, calculated by summation of adrenaline and noradrenaline instantaneous doses) are presented in Table 3 and are illustrated in Figure 4. This support was guided by repeated bedside echocardiographic examination, as previously described [7]. Table 3 summarizes the haemodynamic data recorded at the end of the observational period for both groups. The arterial oxygen tension/inspired fractional oxygen ratio was significantly lower in group 2. Blood lactate level at the end of the observational period and vasoactive support, based on catecholamine dosage, were also significantly greater in group 2.

Rapid improvement in circulatory status was observed in 36 of the 40 group 1 patients, heralded by a significantly lower catecholamine requirement after 24 hours of CVVHDF (Figure 4; *P *< 0.001). However, in four cases circulatory failure did not improve during CVVHDF, circulatory status worsened in the hours following the end of the first session, and complete withdrawal from vasoactive support was never possible. These four patients died after an average of 4.5 ± 2.4 days of haemodynamic support with a vasoactive agent. In the 36 remaining patients complete withdrawal of vasoactive support was possible at the end of the first session, or it could be withdrawn gradually over the following days. Twenty-eight patients ultimately recovered, after 28 ± 19 days of respiratory support. Eight patients later died from recurrence of circulatory failure (*n *= 4) or from a lethal neurological complication (*n *= 4), including an intracranial haemorrhage in one patient. A lower catecholamine requirement during CVVHDF was not observed in group 2 patients (Figure 4). Conversely, the need for catecholamines, which was unchanged or increased during CVVHDF, increased during the hours following the first session, and all the patients in this group died, after an average of 3.5 ± 2.5 days of haemodynamic support with a vasoactive agent.

### Acute renal injury

In this group of 60 patients with refractory septic shock, acute renal injury was defined as diuresis below 30 ml/hour during the observational period of 6–12 hours. In 12 surgical patients, initially managed during the period of observation in the surgical intensive care unit of our hospital, intravenous administration of 500–1,000 mg furosemide failed to increase diuresis. Average plasma urea concentration at the end of the observational period was 16 ± 11 mmol/l and the average serum creatinine was 248 ± 141 μmol/l. There was no significant difference between groups in plasma urea concentration (18 ± 12 mmol/l in group 1 versus 14 ± 8 mmol/l in group 2) or in serum creatinine (255 ± 140 μmol/l in group 1 versus 241 ± 148 μmol/l in group 2).

**Figure 5:**
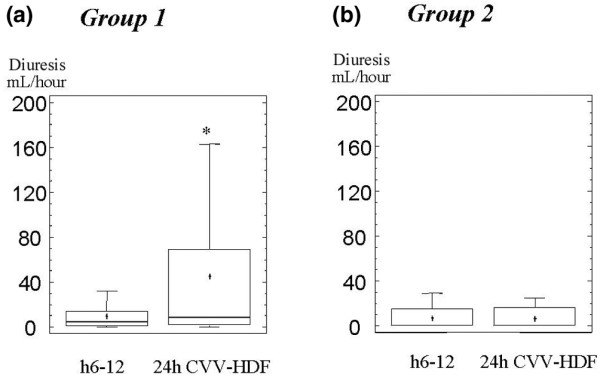
Changes in diuresis. Shown are box and whisker plot analysis (median = horizontal line inside the box; mean = point inside the box) of change in diuresis during the first 24 hours of CVVHDF in **(a) **group 1 (responders) and **(b) **group 2 (nonresponders). A significant increase in diuresis was observed on average in group 1 (*P < 0.001). CVVHDF, veno-venous haemodiafiltration.

A rapid improvement in renal function was observed on average in group 1 patients, heralded by resumption of diuresis after 24 hours of CVVHDF (Figure 5). In the four group 1 patients who died without any substantial improvement in circulatory status, renal function did not improve. In contrast, complete recovery of renal function was observed in 18 patients at the end of the first session. In 18 other patients renal function improved more slowly, and one (*n *= 9), two (*n *= 6), or three (*n *= 3) additional CVVHDF sessions were required before complete renal recovery occurred. During the second session, a negative fluid balance of 5.6 ± 2.9 l (range 1.9–12.2 l) was obtained in these still anuric patients, without any haemodynamic support. Three patients, who remained anuric after three or four CVVHVD sessions, required additional supportive treatment by IHD. Ultimate recovery of renal function was achieved in two cases, but one patient unfortunately died from a neurological complication before renal recovery. Conversely, group 2 patients did not exhibit any improvement in renal function during the procedure (Figure 5). Finally, no blood infusion was required for bleeding related to CVVHDF.

### Additional therapies

Seventeen patients also received corticosteroid treatment. Mortality in this specific subgroup was 53%, which was no different from that for the whole group (53%).

## Discussion

Septic shock is a condition in which renal perfusion is markedly impaired [13], producing acute renal injury [6]. Use of noradrenaline to maintain arterial pressure classically worsens renal hypoperfusion in the low output state [6], but it may improve this perfusion in the hyperdynamic state, a frequent pattern in septic shock [14]. However, when shock becomes refractory, an acute renal failure syndrome may also develop [6]. Even if it is essentially of prerenal origin, renal function is stopped and renal replacement therapy appears logical. Unfortunately, conventional IHD frequently worsens circulatory status in haemodynamically unstable patients, and renal replacement therapy by this method is usually delayed, pending circulatory improvement [4]. Moreover, conventional IHD is usually considered only when metabolic disorders associated with organic renal failure are marked [15], which is not usually the case after an interruption to diuresis of only 6–12 hours. Both of these conditions impose an obligatory delay, during which most patients with refractory shock die.

An important finding of the present study was that a rapid metabolic improvement occurred during early CVVHDF in 67% of patients with refractory septic shock (group 1). Of course, the metabolic acidosis observed in our patients, which was associated with an increased lactate level, was interpreted as mainly resulting from circulatory failure. It was thus considered a consequence, not a cause, of circulatory failure. However, as indicated by abnormally high values of plasma phosphate, prerenal failure contributed in part to the metabolic acidosis. Thus, despite a short duration of renal impairment, renal acidosis was actually present. However, the metabolic improvement observed in group 1 did not appear to result mainly from the buffering action of CVVHDF, because it was associated with a circulatory improvement, permitting a rapid reduction in vasoactive support. This finding is at variance with clinical observations made during IHD. In a recent clinical report, despite adherence to practice guidelines, conventional IHD was associated with a drop in arterial pressure and with increased need for catecholamines in the majority of patients [16].

Concerning the evolution of renal function, the haemodynamic improvement observed during the first CVVHDF session in group 1 patients was associated with rapid renal recovery in half of the patients, as expected with a renal failure of prerenal origin. In the remaining patients from this group, however, this recovery was more progressive, suggesting that some tubular necrosis might have occurred. In these latter cases, additional CVVHDF sessions were required because of delayed recovery of renal function. Moreover, these additional sessions permitted rapid removal of the large volume of fluid given for resuscitation once circulatory failure had been corrected. This beneficial result is impossible with IHD, as was recently illustrated by the report cited above [16], in which only minimal reduction in body weight could be achieved at the end of renal replacement therapy. In three cases, in whom renal recovery was markedly delayed, renal replacement therapy was completed by IHD, which is a perfectly safe procedure when temporally distant from the initial haemodynamic problems.

Another important finding was that lack of metabolic improvement after 12 hours of CVVHDF (for example, unchanged base deficit despite the buffering action of CVVHDF) was associated with a 100% mortality rate. This finding of failure to improve metabolically after 12 hours might be important because it suggests that application of CVVHDF might be futile in such cases. Conversely, the lack of metabolic improvement after 12 hours of CVVHDF might prompt use of drotrecogin alfa, an expensive but efficient treatment for septic shock [17]. The lack of improvement in base deficit after 24 hours of active treatment was recently underscored as a strong predictor of mortality in critically ill patients [18], suggesting the importance of this dosage in the monitoring of septic shock. In that report, a base deficit above 2.5 mmol/l or a blood lactate level greater than 1 mmol/l after 24 hours of adequate support were respectively associated with mortality rates of 71% and 82%.

The particularly poor prognosis of refractory septic shock has led some authors to consider whether strong vasoactive support may be futile in this setting [19]. In this latter report, septic shock initially treated by dopamine and requiring subsequent noradrenaline infusion was associated with a 85% mortality rate. In another report [20] septic patients exhibiting at least three organ failures, as did our patients, had a mortality rate of 92%. The duration of lactic acidosis was found to be a good predictor of multiple organ failure [21], and early lactate clearance was associated with an improved outcome from septic shock in another study [22]. In this context, our strategy of using early CVVHDF appeared to represent a rescue procedure; the final mortality rate of 53% for the whole group was significantly and markedly lower than that predicted by SAPS II (79%). However, this finding needs confirmation in prospective comparative studies. Despite our somewhat different procedure, including only middle volume haemofiltration, continuous dialysis and use of the same filter throughout the session, our results are similar to those recently reported by Honore and coworkers [23]. In a group of 20 patients with refractory septic shock, whose SAPS II score was in the same range as that for our patients, those authors observed a mortality rate of 55%, in contrast to the predicted mortality of 79%.

Our present data suggest that early renal replacement therapy by CVVHDF might have a beneficial effect as an adjunctive treatment for refractory septic shock. This therefore raises a major question about the precise mechanism by which CVVHDF may improve this condition. Many humoral mediators that are potentially involved in the inflammatory response associated with sepsis are 'middle molecules', which are cleared by the technique. For this purpose, convection has been proved to be more efficient than diffusion [24]. However, recent studies have yielded conflicting results. De Vriese and coworkers [25] demonstrated effective cytokine removal in patients with septic shock, but in a recent phase II randomized study Cole and coworkers [26] were unable to confirm that early use of haemofiltration at a filtration rate of 2 l/hour reduced the circulating concentration of cytokines associated with septic shock. Increasing the rate of fluid exchange across the membrane will increase convective transport, and this led the same group of investigators to suggest that high-volume haemofiltration might improve clearance of mediators in refractory septic shock [27]. In the present study we did not use high-volume haemofiltration, as defined by Ronco and coworkers [28] as a threshold of 35 ml/kg per hour. Use of 2,000 ml/hour haemofiltration in our group of patients, whose average body weight was 72 kg, would have resulted in an average convection exchange of 28 ml/kg per hour. However, in a recent study Cole and coworkers [27] compared high-volume versus standard-volume haemofiltration in a group of 11 septic patients, and found that both techniques lowered the plasma concentration of mediators. The kinetics of this decrease suggested that it mainly resulted from membrane adsorption, which was achieved with both techniques, and in a recent pro/con debate the benefit of high-volume haemofiltration was challenged [29]. Thus, membrane adsorption might be responsible, at least to some extent, for the haemodynamic improvement observed in the present study.

However, early and adequate renal replacement therapy might also have contributed to the improved prognosis. Acute renal failure *per se *exerts an independent and specific effect on the morbidity of critically ill patients [30,31]. The mortality rate in acute renal failure occurring in a septic context was 75% in a large prospective study, in which IHD was the quasi-exclusive therapeutic procedure [32]. Critically ill patients probably benefit from a combination of diffusion and convection that provides sufficient elimination of small and larger toxins and, in contrast to the majority of other reports, we combined dialysis with our procedure, anticipating better treatment of renal failure syndrome [33]. Also, this technique probably permitted a more rapid correction of metabolic acidosis by acting on the renal component of this disorder. However, if it should be considered, any impact of this choice on the clinical outcome remains purely hypothetical.

## Conclusion

Without providing any idea regarding the precise mechanism, which would require additional comparative studies, our clinical report suggests that early renal replacement therapy by CVVHDF may improve the prognosis of the most severe forms of septic shock, and should be considered as an adjunctive therapy in sepsis-related multiple organ failure. Moreover, after 12 hours, this procedure distinguishes a subgroup of patients with a 100% probability of death and so it could perhaps help in deciding whether to institute a more expensive treatment, such as drotrecogin alfa.

## Key messages

• 		Early CVVHDF may improve the prognosis of sepsis-related multiple organ failure.

• 		Failure to correct metabolic acidosis rapidly during CVVHDF (for example nonresponders) is a strong predictor of mortality.

• 		By screening nonresponders, early CVVHDF could help in deciding whether to institute a more expensive treatment, such as drotrecogin alfa.

## Abbreviations

CVVHDF = continuous veno-venous haemodiafiltration; IHD = intermittent haemodialysis; SAPS = Simplified Acute Physiology Score.

## Competing interests

The authors declare that they have no competing interests.

## Authors' contributions

FJ and AV-B conceived and designed the study, and drafted the manuscript. FJ supervised and was responsible overall for all aspects of the study. BP acquired a substantial proportion of the data. KC, OP and AR performed data collection. AB and PA supplied statistical expertise.

## Figures and Tables

**Table 1:** Comparison of physiological data between group 1 and group 2.

**Parameter**	Group 1 (n = 40)	Group 2 (n = 20)	*P* value
Age (years)	56 ± 16	58 ± 16	0.56
SAPS II score	61 ± 13	72 ± 21	0.08
LODS score	13 ± 1.8	14.6 ± 3.2	0.02
McCabe score (n [%])			
0	31(77)	15 (82)	1
1	9 (23)	5 (28)	
Immuno-incompetent (n [%])	8 (20)	7 (35)	0.34
Shock (n [%])			
Primary	28 (70)	16 (80)	0.61
Secondary	12 (30)	4 (20)	
Reason for admission (n [%])			
Medical	29 (72)	16 (80)	0.75
Surgical	11 (26)	4 (20)	
Aetiological agent identified (n [%])			
Yes	31 (78)	11 (55)	0.14
No	9 (22)	9 (45)	
Corticosteroid therapy (n [%])	12 (30)	5 (25)	0.92
Mortality (%)			
Predicted	67	87	<0.0001
Observed	30	100	

In group 1 (responders), there were 22 cases of bacterial pneumonia and eight cases of sepsis of extrapulmonary origin among medical patients, and 10 cases of peritonitis among surgical patients. In group 2 (nonresponders) there were 11 cases of bacterial pneumonia and seven cases of sepsis of extrapulmonary origin among medical patients, and two cases of peritonitis among surgical patients. No significant difference was found between these distributions. LODS, Logistic Organ Dysfunction Score; SAPS, Simplified Acute Physiology Score.

**Table 2:** Average plasma electrolyte concentrations before CVVHDF

Electrolytes	Group 1 (*n *= 40)	Group 2 (*n *= 20)
Cations (mEq/l)		
Sodium	134 ± 5	135 ± 4
Potassium	4 ± 1	5 ± 1*
Anions (mEq/l)		
Chloride	98 ± 6	98 ± 4
Bicarbonate	17 ± 5	16 ± 5
Phosphate	1.9 ± 0.9	2.6 ± 0.9 *
Protein	12 ± 3	12 ± 4

*P < 0.05. CVVHDF, veno-venous haemodiafiltration.

**Table 3:** Blood gas analysis and haemodynamic parameters at the end of the 6-hour observational period

Parameter	Group 1 (*n *= 40)	Group 2 (*n *= 20)	*P *value
PaO_2_/FiO_2 _(mmHg)	139 ± 68	100 ± 68	0.046*
PaCO_2 _(mmHg)	49 ± 11	53 ± 12	0.18
BE (mmol/l)	-11 ± 4	-13 ± 5	0.10
pH	7.17 ± 0.11	7.11 ± 0.13	0.06
Lactate (mmol/l)	4.3 ± 1.8	5.8 ± 3.2	0.020*
HR (beats/minute)	118 ± 20	113 ± 20	0.48
CI (l/minute per m^2^)	3.2 ± 1.2	2.9 ± 1	0.39
LVEF (%)	50 ± 16	48 ± 19	0.73
Vasopressor support (choice; *n *[%])			
Noradrenaline	19 (48%)	9 (45%)	0.97
Noradrenaline + dobutamine	7 (18%)	4 (20%)	
Adrenaline	14 (35%)	7 (35%)	
Vasopressor support (dosage; μg/kg per minute)	1.1 ± 0.8	2.3 ± 1.4	0.002*

Shown is a comparison of blood gas analysis and haemodynamic parameters at the end of the 6-hour observational period between group 1 (responders) and group 2 (nonresponders). Vasopressor dosage is the cumulative dosage of major catecholamines (noradrenaline [norepinephrine] or adrenaline [epinephrine]), with dobutamine being given at 5 μg/kg per minute. *Statistically significant finding. BE, base excess; CI, cardiac index; FiO_2_, fractional inspired oxygen; HR, heart rate; LVEF, left ventricular ejection fraction; PaCO_2_, arterial carbon dioxide tension; PaO_2_, arterial oxygen tension.
